# Can ploidy changes propel the evolution of allogamy in a selfing species complex?

**DOI:** 10.1186/s12870-025-06868-1

**Published:** 2025-08-01

**Authors:** Ana García-Muñoz, Camilo Ferrón, Celia Vaca-Benito, Carlos Olmedo-Castellanos, María Nazaret Martínez-Gómez, Tatiana López-Pérez, Mohammed Bakkali, Sílvia Castro, Mariana Castro, João Loureiro, A. Jesús Muñoz-Pajares, Mohamed Abdelaziz

**Affiliations:** 1 BioChange Network, Departamento de Genética, Universidad de Granada, Granada, 18071 Spain; 2 Departamento de Biología y Geología, Física y Química Inorgánica, Universidad Rey Juan Carlos (URJC), Móstoles, 28933 Spain; 3 Instituto de Investigación en Cambio Global (IICG-URJC), Universidad Rey Juan Carlos, Móstoles, 28933 Spain; 4 Departamento de Genética, Universidad de Granada, Granada, 18071 Spain; 5 Centre for Functional Ecology, Associate Laboratory Terra, University of Coimbra, Coimbra, Department of Life Sciences, Coimbra, 3005-531 Portugal; 6 Research Unit Modeling Nature, Universidad de Granada, Granada, 18071 Spain

**Keywords:** *Erysimum incanum*, Herkogamy, Inbreeding depression, Pollen:ovule ratio, Self-pollination

## Abstract

**Background:**

The transition to self-fertilization has occurred repeatedly across diverse plant groups, and the evolutionary consequences of selfing typically suggest that a shift toward outcrossing is unlikely. However, we propose that polyploidization could drive changes in reproductive strategies by influencing traits associated with pollination. We explored various traits linked to the mating system across different ploidy levels within the polyploid *Erysimum incanum* species complex, which is generally considered a predominantly selfing species.

**Results:**

Our results revealed significant variation in self-fertilization success across different ploidies and we also found significant differences among populations within the same ploidy level. Inbreeding depression is absent in diploids, it was present in hexaploids, while tetraploids exhibited intermediate values. Additionally, polyploids showed traits more commonly associated with outcrossing rather than self-fertilization. Finally, the high values of heterozygosity found in polyploid populations were contrary to our expectations due to the selfing evolutionary history of this species.

**Conclusions:**

These findings suggest that polyploidy may facilitate the emergence of alternative reproductive strategies, driving diversification in mating systems within this selfing species complex. This phenomenon, not previously observed in the wild, opens new perspectives on the evolution of plant mating systems.

**Supplementary Information:**

The online version contains supplementary material available at 10.1186/s12870-025-06868-1.

## Background

The evolution of mating systems in plants and the mechanisms driving their diversity in nature have captivated scientists since Darwin [47, 74]. Most flowering plants are hermaphrodites, producing both pollen and ovules within the same flower [22]. However, they exhibit impressive diversity in reproductive strategies [14], including changes in floral organs to optimize mating success [7, 78, 81]. Mating systems are shaped by a wide range of self-incompatibility levels, varying from self-compatible specie, which often shows autonomous or assisted self-pollination, to highly self-incompatible taxa that relies on cross-fertilization, including intermediate stages between both extremes [115]. While there are intermediate states between selfing and outcrossing strategies, known as mixed mating systems [62], the bimodality of mating systems in plants [115] persists because shifts from outcrossing to selfing occur regularly [86, 146]. This frequent transition is accompanied by changes at the phenotypic level that comprise the well-known ‘selfing syndrome’ [41].

Reproductive assurance, an evolutionary consequence of selfing, provides independence from mate availability and/or pollination vectors, granting opportunities for colonization [23]. From a genetic perspective, self-fertilization alleles are expected to spread in the population, as selfers pass their entire genetic makeup to their offspring [54, 68]. However, the advantages of selfing can be countered by the fitness reductions experienced by inbred offspring, i.e. inbreeding depression [32]. This occurs when recessive deleterious alleles become unmasked as homozygote frequency increases [31]. In this context, deleterious alleles may be purged from the population by the action of natural selection [31, 70, 84, 120],but see [24]. The reduced genetic diversity exhibited by selfing populations [5, 83] would be detrimental for coping with environmental unpredictability and, thus, may constrain their evolutionary outcomes, however, the assumption of a strong reduction of genetic diversity is not supported by other models [37]. On the other hand, the heterozygosity generated by outcrossing can create new allelic combinations [77, 97] that lead to heterosis events [40, 123]. Since recessive deleterious alleles have not been purged, outcrossers commonly experience inbreeding depression after self-fertilization [92, 94].

Mating system transitions have significant causes and consequences for genetic structures [108, 125] and the evolutionary dynamics of populations [8, 33, 141]. However, the transition between mating systems is asymmetric: outcrossers seldom evolve from selfers, since the accumulation of deleterious alleles (i.e. inbreeding depression) is effectively purged in selfing species as a result of purifying selection, and they also exhibit significant genetic and ecological advantages that affect their reproduction [72]. The lower speciation and higher extinction rates in selfing lineages may represent an evolutionary dead end (SEDE hypothesis) [132]. This hypothesis has been supported and well-documented by numerous examples of independent evolution of selfing, which has led to a broad understanding of the evolutionary consequences of this transition [30, 31, 63, 129, 132]. The reverse transition—from selfing to outcrossing—has been considered theoretically unfeasible due to the lack of documented evidence and examples in nature,nevertheless, clear evidence is still lacking [72, 132]. The long-term maintenance of mixed mating systems is also controversial, as they are often viewed as an intermediate step in the transition from outcrossing to selfing [60].

The pollen:ovule ratio (P:O ratio) has been used as a conservative indicator in mating system characterization [42]. According to sex allocation theory [34, 35], high values of this index are expected in outcrossing species to compensate for pollen losses during transport. In contrast, this ratio is lower in selfing species, as there is no inter-individual pollen transfer. The P:O ratio is often correlated with other floral traits, such as floral size and herkogamy [57], suggesting that the functions of these traits are closely related [91, 130], and connected to the plant’s mating strategy. However, the P:O ratio also exhibits inter-population and inter-individual variability [43], which can be explained by environmental factors influencing outcrossing and selfing rates. These factors contribute to mating system variation among locally adapted populations and even among years within the same population [36, 100]. Variation in mating systems has important macro- and microevolutionary consequences due to changes in population genetics and interactions with other species [33].

Mating systems are shaped by traits related to pollination and plant reproduction, including both primary and secondary sexual traits [16, 109]. For example, corolla size is one of the most representative traits related to plant attractiveness, enhancing pollinator visitation and pollen exportation [27, 59, 67]. A proper relative position of male and female sexual organs is crucial to facilitate their contact in selfing plants. However, the co-occurrence of both sexual organs can become problematic in plants with significant inbreeding depression. In this context, the separation of anthers and stigma, known as herkogamy, is a pivotal trait to avoid sexual conflict and mitigate the consequences of inbreeding depression [11, 32]. Similar to the P:O ratio, such traits are also used to infer the predominant reproductive strategy. Genetic factors influence the variation in these traits and, consequently, the evolution of mating systems [44, 51, 79, 80].

Polyploidy, a genetic condition that has become widespread in flowering plants [137], has significant implications for plant phenotype, physiology, and biotic interactions [110, 122]. Polyploidization, which results from whole genome duplications or hybridization events, has the potential to increase genetic diversity through the higher frequency of recessive mutations allowed by higher heterozygosity levels in polyploids [38, 39, 112],or by the contribution of non-additive genetic effects [102, 138]. While the effects of ploidy on plant phenotype are well understood [101, 135], its influence on mating system evolution remains unclear. Traditionally, it has been suggested that polyploid lineages exhibit reduced inbreeding depression, which may explain the colonization ability of polyploids in selfing species [15, 18, 70, 84, 117, 126, 133].

Although there is no conclusive evidence [96], the association between polyploidy and self-fertilization has been hypothesized [18] and extensively documented in certain groups such as Brassicaceae [50]. The breakdown of self-incompatibility (SI) systems due to genome duplication [96] and/or the avoidance of the minority cytotype disadvantage [85] are hypothesised to be the underlying mechanisms of this association [18]. However, exhaustive studies exploring mating system variation across multiple ploidy levels are lacking [28, 29]. Only a few experimental studies have compared populations differing in both ploidy and mating systems [71, 76, 96, 99, 118], while studies addressing this question in populations with the same mating system are even scarcer [19]. Given the intra-specific variation in reproductive strategies and mating traits, considering multiple populations has become increasingly important for accurate mating system characterization [140]. In fact, inbreeding depression estimates have been shown to vary among populations [19], and this variation could be more pronounced if populations differ in ploidy level.

In this study, we examined the polyploid *Erysimum incanum* species complex, which includes a majority of selfing species [53, 106] and is expected to have reduced genetic diversity due to its evolutionary history. We explored the relationship between ploidy and reproductive strategy by measuring traits closely related to the mating system. Specifically, we compared mating system traits—such as inbreeding depression, reproductive investment (including the P:O ratio), and floral size—among different taxa of the *Erysimum incanum* species complex. The primary aims of this study were: (i) to assess the ploidy levels of the studied populations of the *E. incanum* species complex, (ii) to infer the mating system of each taxon by measuring various mating system traits, (iii) to explore variation in secondary reproductive traits across ploidy levels and populations, and (iv) to investigate genetic differences within the group. These approaches provide insight into potential mating system diversification within this group.

## Methods

### Study system

The genus *Erysimum* L. is one of the most diverse in the Brassicaceae family, with species found throughout Eurasia, North and Central America, and North Africa [9]. Diversification within the genus has been driven by patterns of local adaptation and hybridization between lineages [2, 103]. *Erysimum incanum* is considered a species complex that includes annual and monocarpic species and subspecies distributed across the eastern Iberian Peninsula, southeastern France, and the four major mountain ranges in Morocco [4, 53, 107]. This complex contains both diploid (2n = 2x = 16 chromosomes) and tetraploid (2n = 4x = 32 chromosomes) populations [53, 107]. Evidence about the origin of polyploidy in this genus remains currently unclear. Diploid populations of *E. incanum* are found in the Rif and Pyrenees mountains, where *E. incanum* subsp. mairei and *E. aurigeratum* were described, respectively. The tetraploid populations consist of *E. incanum* subsp. *incanum*, which has a vicariant distribution in the Iberian Peninsula and Morocco [53, 107]. *E. incanum* subsp. *incanum* in Morocco is found in the Middle Atlas, High Atlas, and Anti-Atlas Mountains [53, 107]. The selfing syndrome in the *Erysimum incanum* species complex is characterized by small, hermaphroditic, and self-compatible flowers [106], with anther rubbing mechanisms that promote self-pollination [1]. In this study, we analyzed nine populations of *E. incanum*, with geographic origins detailed in Table 1. Dr. Juan Lorite identified the plant material used in this study. Voucher specimens are deposited in University of Granada Herbarium (Ei08: GDA56843-1; Ei09: GDA8844-1; Ei11: GCA60084-1; Ei13: GDA28250-1; Ei17: GDA75378; Ei19: GDA75377), Institut Botànic de Barcelona herbarium (Ei16: BC138876) and University of Barcelona Herbarium (Ei12: BCN91759; Ei18: BNC060125).

**Table 1:** Summary of the populations (Pop.), their geographical location and coordinates and the number of individuals and crosses included in this study

Ploidy	Pop	Location	No. plants	No. crosses	G.S	ID	99% Bootstrap Intervals
2x	Ei12	Rif 35º11.14’N, 5º13.32’W	67	196	0.35 ± 0.01	−0.310	[−0.317,−0.302]
2x	Ei13	Pyrenees 41º58.34’N, 0º46.72’W	19	34	0.34 ± 0.01	−0.399	[−0.469,−0.331]
4x	Ei08	Mid Atlas 33º27′06’’N, 4º57′38’’W	302	4013	0.78 ± 0.10	−0.009	[−0.014,−0.005]
4x	Ei09	Baetic 37º20′18’’N, 3º02′38’’W	110	572	0.78 ± 0.03	−0.125	[−0.162,0.088]
4x	Ei11	Baetic 37º48′39’’N, 1º47′41’’W	46	203	0.84 ± 0.07	−0.111	[−0.121,−0.101]
6x	Ei16	High Atlas 30º50′04’’N, 8º23′38’’W	90	527	1.10 ± 0.03	0.043	[0.017,0.070]
6x	Ei17	High Atlas 31º17′36’’N, 7º13′57’’W	25	641	1.12 ± 0.04	0.562	[0.553,0.571]
6x	Ei18	High Atlas 30º14′16’’N, 8º10′37’’W	74	543	1.13 ± 0.03	0.108	[0.098,0.118]
6x	Ei19	High Atlas 29º48′08’’N, 8º45′34’’W	26	246	1.11 ± 0.02	−0.390	[−0.397,−0.384]

The genetic information of each population is given by the genome size (G.S. mean ± SD) and the ploidy level (Ploidy). Inbreeding depression (ID) was calculated for the predispersal fitness components (seed fertilization and mature seed formation). The significance of ID was estimated by calculating 99% confidence interval by bootstrapping

### Chromosome analysis and genome size measurements

For chromosome analysis, we used material from one of our works in preparation (Abdelaziz et al., In prep.), where root meristems were obtained from germinated seeds sampled in the natural populations specifically prepared for this purpose. Using a protocol modified from Inceer and Beyazoglu [73], the meristems were separated from the rest of the root and treated with 0.05% colchicine for one hour. Subsequently, the roots were fixed in a 3:1 ethanol-acetic acid solution for 24 h at 4 °C. Finally, they were stained with 3% aceto-orcein for 12 min and visualized under an Olympus BX41 microscope using a 100X objective and the pictures were taken with an Olympus DP70 camera.

Holoploid genome size analyses (2C in pg; sensu [64]) were carried out as described by Muñoz-Pajares et al. [104]. Briefly, 50 mg of *Erysimum* leaf tissue and 50 mg of an internal reference standard (*Solanum lycopersicum* ‘Stupické’, 2 C = 1.96 pg; [49]) leaves were co-chopped in a Petri dish with 1 ml of Woody Plant Buffer (WPB, [87]). The nuclear suspension was filtered, and 50 µg/mL of propidium iodide (PI, Fluka, Buchs, Switzerland) and 50 µg/mL of RNAse (Fluka, Buchs, Switzerland) were added. After 5 min, samples were analysed using a Sysmex CyFlow Space flow cytometer (Partec GmbH, Görlitz, Germany) and results were acquired with Sysmex FloMax software (v. 2.5). Holoploid genome size was calculated by multiplying the DNA index (ratio between mean FL of sample and standard G1 nuclei) by the nuclear DNA content of the reference standard, and the DNA ploidy level was estimated [104] Between 10 and 20 individuals per population were analyzed from each range where these species occur. Populations with homogeneous ploidy levels were considered to consist primarily of a single cytotype.

### Crossing experiment

Flowers from each population were subjected to two controlled hand-pollination treatments: (1) Selfing, where pollen from the same flower was deposited onto the style, and (2) Intra-population Outcrossing, where flowers were pollinated with pollen from another individual within the same population. To prevent self-pollination before the treatments, flowers were carefully emasculated just before opening. Donor and receptor plants were at similar stages of flowering. Plants were not separated according to the type of treatment, so both types of crosses could be conducted in different flowers from the individual. Crossed flowers were not used for phenotyping (see next section) to avoid the effects of manipulation in the rest of measurement. In total, 6595 selfing and 380 outcrossing crosses were conducted on 759 *E. incanum* plants, with a maximum of 140 self-pollinated and 10 cross-pollinated flowers per individual. The number of individuals and crosses for each population is detailed in Table 1.

Fruits were collected to count the number of filled seeds, aborted seeds, and non-fertilized ovules. Three post-pollination fitness components were calculated and compared between *E. incanum* ploidy levels as a proxy for autonomous selfing success. Seedset refers to the proportion of filled seeds produced per fruit relative to the total number of ovules (the sum of filled seeds, aborted seeds and unfertilized ovules), while fertility estimates the proportion of fertilized ovules (filled seeds and aborted seeds) compared to the total number of ovules per fruit. A third fitness component, fertility success, measures the proportion of filled seeds relative to the number of fertilized ovules. The seedset for a treatment was considered zero if the fruit did not develop. These fitness components result from pollen-stigma-ovule interactions, which may fail at two stages: (1) before fertilization, due to the inhibition of pollen tube growth, preventing fertilization, and (2) after fertilization, when fertilized ovules fail to form fully developed seeds. To explore this, we conducted preliminary in vivo visualization of pollen tube growth using UV microscopy, following a modified aniline blue staining protocol from Xie et al. [142]. Self-pollinated flowers were collected 72 h after pollination and preserved in 90% alcohol. A total of 46 flowers from the three ploidy levels were examined.

The relative reproductive success of selfed versus outcrossed flowers is referred to as inbreeding depression. This can be quantified for various fitness components, ranging from pre-dispersal to post-dispersal stages, such as germination, growth, and offspring reproduction. In this study, we focused on pre-dispersal fitness, which includes seed fertilization and the formation of mature seeds. The inbreeding depression coefficient (ID) was calculated using seedset data from selfing and outcrossing treatments, for each individual plant, population, and ploidy level, following Ågren & Schemske [6]:

$$\mathrm{ID}=1-{\mathrm w}_{\mathrm s}/{\mathrm w}_{\mathrm o}\;\mathrm{if}\;{\mathrm w}_{\mathrm s}\;\leq{\mathrm w}_{\mathrm o}$$

$$\mathrm{ID}\;={\mathrm w}_{\mathrm o}/{\mathrm w}_{\mathrm s}-1\;\mathrm{if}\;{\mathrm w}_{\mathrm s}\;>{\mathrm w}_{\mathrm o}$$

In this study, ws represents the fitness component (seed set) from the selfing treatment, while wo represents the seed set from the outcrossing treatment. Inbreeding depression (ID) can range from −1 to 1. Negative and significantly different from zero values indicate outbreeding depression, meaning selfing offspring have higher fitness than outcrossed ones [56]. Significant positive values indicate inbreeding depression, where outcrossing offspring show superior fitness compared to selfed offspring. The significance of these values was calculated using 99% bootstrap confidence intervals, based on 10,000 permutations using the *boot* package (version 1.3–28) in R [25]. Values not significantly different from zero indicate no fitness difference between selfed and outcrossed offspring.

### Mating traits measurement

We took additional measurements in an independent group of 386 individuals (101 diploids, 144 tetraploids and 141 hexaploids) that were grown simultaneously in the same conditions. For one flower in anthesis from each plant, we measured the following phenotypic traits: corolla diameter (i.e., the distance between the edges of opposite petals), the length of the long stamens (i.e., the distance from the base of the long filament to the anther), and the length of the style (i.e., the distance from the insertion point of the style at the base of the corolla tube to the stigma surface). Short anthers were excluded from analysis as they do not contribute to spontaneous selfing. Herkogamy was calculated as the difference between stamen length and style height, with positive values indicating that the stigma is positioned above the stamens, and negative values indicating the stigma is below the stamens, facilitating pollen drop onto the stigma. Anther exertion was also estimated as the distance between the anthers and the corolla surface, as this exposure potentially influences pollen dispersal. These flowers had not been previously subjected to the crossing experiments in order to avoid manipulation effects on the measurements.

Additionally, we collected half of the stamens (two long and one short stamen) from these flowers, preserving them in 70% alcohol for pollen grain counting. We counted the pollen grains of the collected stamens using the Multisizer Coulter Counter 3 particle counter (Beckman Coulter, Pasadena, CA, USA) provided by the morphometric lab of the Centro de Investigación, Tecnología e Innovación (CITIUS) at the University of Seville (Spain). To get a proxy for the total pollen in each flower, we multiplied this value by two.Male and female reproductive investments were estimated by calculating the total pollen and ovule production per plant at the end of the plant life cycle. The number of pollen grains and ovules per flower was multiplied by the total number of flowers produced. These values were used to calculate the P:O ratio described by Cruden [42].

### Genetic diversity estimation

We obtained whole-genome sequencing data from one individual per studied population. Leaf material was collected and preserved in silica gel for DNA extraction, using the GenElute™ Plant Genomic DNA Kit (SIGMA), following the manufacturer’s instructions. DNA purity, integrity, and concentration were assessed using agarose gel electrophoresis, as well as spectrophotometric methods (Nanodrop and Qubit). Individual Illumina libraries were prepared using the Collibri ES DNA Library Prep Kit (ThermoFisher) and sequenced by Novogene using the NovaSeq sequencing system. After quality control of paired-end raw reads with FastQC (v.0.11.5; [10]), the resulting fastq files were mapped to the *E. cheiranthoides* reference genome (http://erysimum.org/) using BWA (v.0.7.15; [88]). SAMtools (v.1.4.1, [89, 90]) was used to convert SAM files to BAM files, and BCFtools (v.1.10; [46]) was used for variant calling, resulting in 14,571,587 raw SNPs. After filtering for quality (≥ 20), removing missing data, indels, and linkage disequilibrium using VCFTools (v.0.1.17; [45]) and PLINK (v.1.90b5.2 [114],), 2,354,696 SNPs remained for further analysis. The proportion of heterozygous sites was calculated using observed and expected heterozygosities (H_O_ and H_E_) with VCFTools’ (–het) function.

### Statistical analyses

Autonomous selfing success, inbreeding depression, and other mating system traits were compared among ploidies and populations using ANOVA and Tukey’s test in R. The combined effect of ploidy and treatment on seed set was explored with a two-way ANOVA. Pearson’s product-moment correlations, performed with R’s Hmisc v5.1–2 package [66], were used to explore relationships between corolla diameter, herkogamy, P:O ratio, and inbreeding depression across ploidy levels. Correlations were represented using the corrplot v.0.95 package [139] in R.

We used generalized linear mixed models (GLMM) implemented in the *lme4* v. 1.1.32 package [21, 147] in R to assess the effect of treatments and ploidy (as fixed factors) and their interaction on fitness components (seed set, fertility, and fertility success). Plant identity was treated as a random factor, and random effects were visualized using caterpillar plots. Model comparisons were made using Akaike’s Information Criterion (AIC), Bayesian Information Criterion (BIC), log-likelihood (LogLik), and chi-squared tests (*χ*^2^). Effect sizes of each model were computed by calculating the R^2^ coefficient [105] with a function available in the MuMIn v.1.48.11 package [20]. All statistical analyses were performed in R Statistical Software (v4.2.1, [134].

## Results

### Genome size

The somatic chromosome numbers (2n) and the ploidy level found for the different populations included in the study were 2n = 16 (2x), 2n = 32 (4x) and 2n = 48 (6x) (Table 1; [Additional File 1] We also identified three distinct, non-overlapping genome size ranges in the studied populations corresponding to the three level of ploidy identified (Table 1). As genome size was consistent across individuals within each population, we assumed that each population was composed of a single cytotype.

### Selfing and outcrossing success

**Fig. 1:**
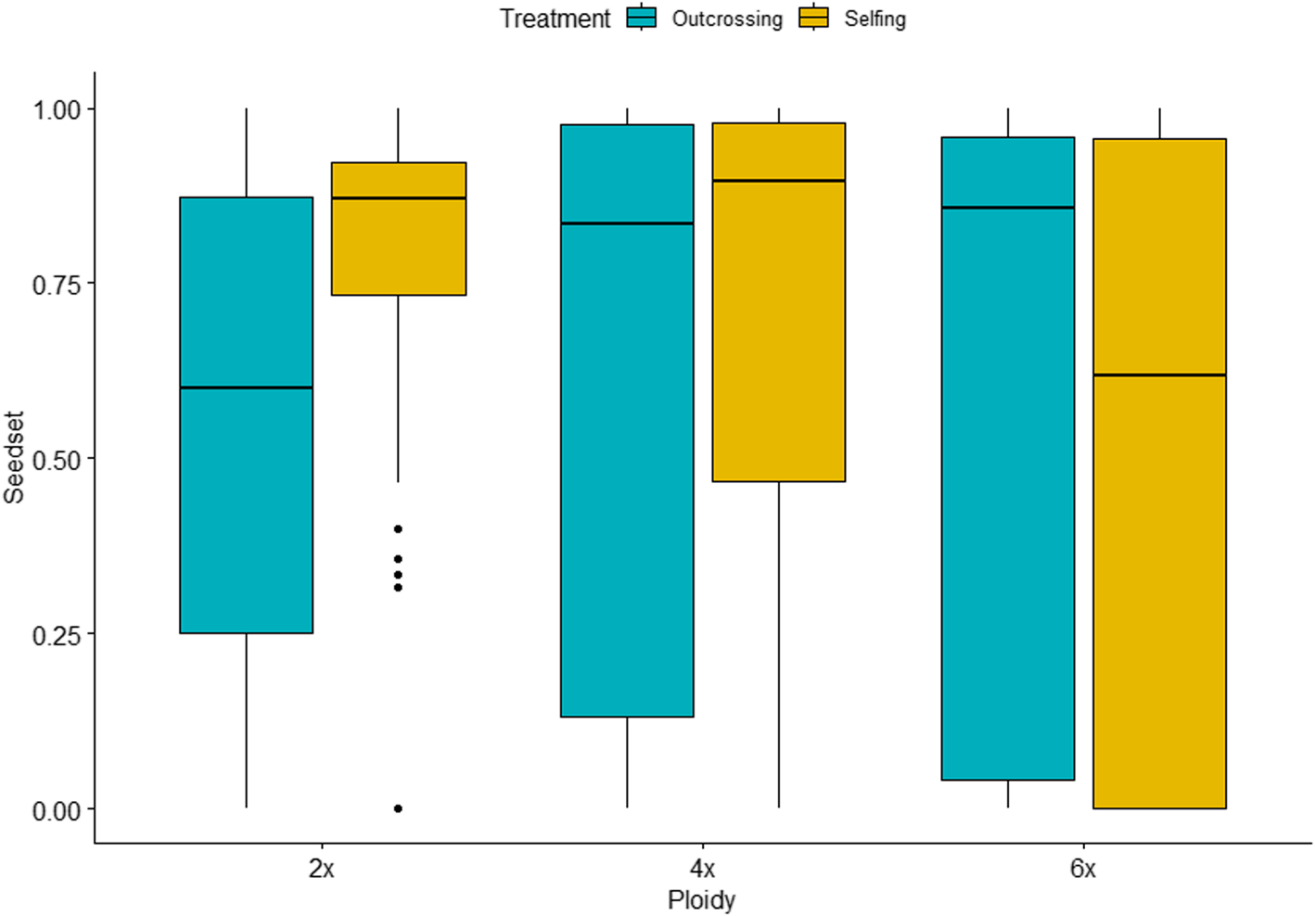
One of the fitness components, estimated as seedset, resulting from selfing and outcrossing treatments for each ploidy level

Pollination treatment (selfing vs outcrossing) significantly influenced the seed set (F = 10.731, *p* < 0.0001, df = 2). Ploidy also had a significant effect on the seed set (F = 77.320, *p* < 0.0001, df = 2). The success of selfing varied more with increasing ploidy, with seed set showing a wider distribution ranging from high to nearly zero values in polyploids (Fig. 1). In contrast, seed set distributions were relatively large in outcrossing treatments, regardless of ploidy (Fig. 1). When treatments were analyzed separately, significant differences in reproductive success from selfing crosses were observed among ploidy levels for all fitness components [see Additional file 2a]. In diploids, fertility values were near 1, indicating that nearly all ovules were fertilized [see Additional file 2a]. However, no significant differences in any fitness components were found across ploidy levels in outcrossing treatments [see Additional file 2b]. GLMM analyses showed that both pollination treatment (selfing or outcrossing) and ploidy level (diploid, tetraploid, or hexaploid) influenced the seed set, with the best model including the interaction between these two factors (Table 2). The same pattern was observed for fertility and fertilization success [see Additional file 3 and Additional file 4].

**Table 2:** Outcome of the GLMM analyses testing the effect of the treatment (*Selfing* or *Outcrossing*) and ploidy as fixed factors together with their interaction on *E. incanum* seedset

		**Seedset**					
**Model**	***Estimate***	**AIC**	**BIC**	**logLik**	***χ***^***2***^	**df**	***p-value***
**Intercept**	0.54	2973.4	2999.3	−1482.7			
**Seedset ~ Treatment + (1 |Population/Plant)**		2970.8	3003.2	−1480.4	4.5	1	< 0.05*
Outcrossing	0.05						
**Seedset ~ Ploidy + (Ploidy |Population/Plant)**		2967.1	3070.8	−1467.5	25.7	11	< 0.01**
* 4x*	−0.24						
* 6x*	−0.38						
Seedset ~ Treatment * Ploidy + (Ploidy |Population/Plant)		2946.3	3609.4	−1453.2	26.8	3	< 0.0001****
* Outcrossing*	−0.42						
* 4x*	−0.25						
* 6x*	−0.42						
* Outcrossing:4x*	0.78						
* Outcrossing:6x*	0.34						

The individual plant and the population were considered as random factors nested within the ploidy level. Significant *p*-values are indicated in bold

Notation from *lme4* package is shown for brevity. Proper mathematical notation corresponding to each model is detailed in Additional File 8

Notably, we observed significant differences in reproductive success from selfing among populations [see Additional file 5a], but no such differences were found for outcrossing treatments [see Additional file 5b]. Variation among populations increased with ploidy. Diploid populations did not differ significantly, while higher differences were observed in tetraploids (though non-significant), and accentuated differences were found among hexaploid populations. Surprisingly, both the highest and lowest seed set values were found within the hexaploid group [see Additional file 5a].

### Inbreeding depression

Inbreeding depression was significant in all populations except the tetraploid *Ei09* population (Table 1). Diploid and tetraploid populations mostly exhibited significant negative inbreeding depression values. These values were closer to zero in tetraploids than diploids, though still significant in 2 out of 3 populations (*Ei08* and *Ei11*; Table 1). In contrast, most hexaploid populations displayed significant positive inbreeding depression, with the exception of the *Ei19* population, which showed values similar to those of diploid populations. When comparing inbreeding depression across ploidies, we found a significant increase in inbreeding depression with increasing ploidy (F = 2257, *p* < 0.0001, df = 2). Diploids exhibited clear outbreeding depression, with a mean value of inbreeding depression of −0.289 [−0.443—0.088], while hexaploids showed the highest levels of inbreeding depression with a mean of 0.183 [0.050–0.293]. The mean value of inbreeding depression found in tetraploids was also negative, −0.094, but closer to zero than diploids. Values of inbreeding depression in tetraploids ranged from −0.242 to 0.031, being intermediated between diploids and hexaploids. The values of inbreeding depression were significantly different between the three levels of ploidy.

### Reproductive investment

**Fig. 2:**
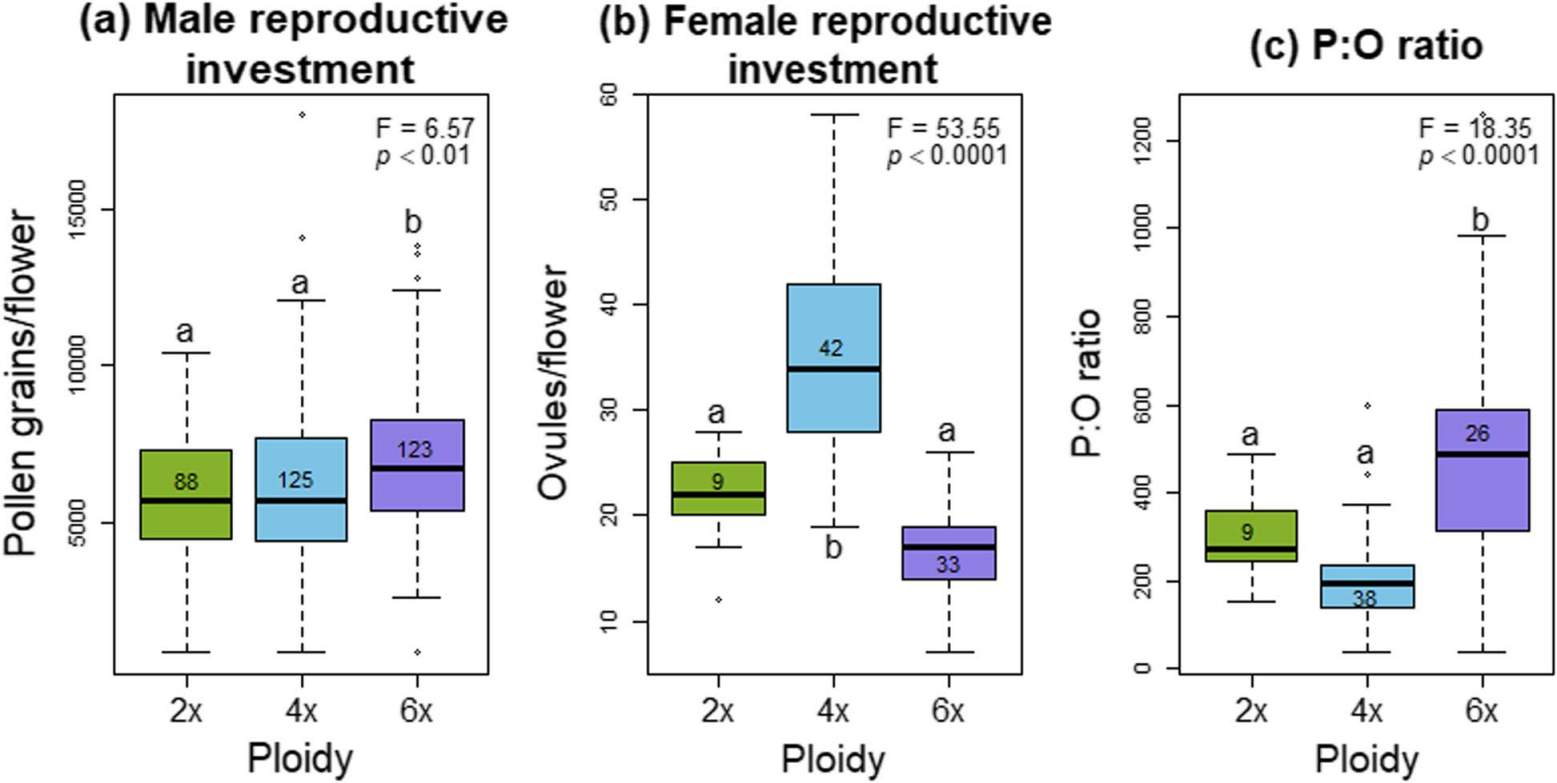
Reproductive investment among ploidies for (**a**) male function measured as pollen production, (**b**) female function measured as ovule amount and (**c**) the relative investment between male and female function estimated as P:O ratio. Different letters indicate significant differences among ploidies according to the Tukey's test. Significance *p*-values indicate the ANOVA results among ploidies

We observed significant differences in both male and female reproductive investment across ploidy levels. Hexaploid plants produced significantly more pollen than diploids and tetraploids (F = 6.57, *p* < 0.01, df = 2), while female reproductive investment (ovule production) was significantly higher in tetraploids (F = 53.55, *p* < 0.0001, df = 2). This resulted in a significantly higher P:O ratio in hexaploid plants compared to diploids and tetraploids (F = 18.35, *p* < 0.0001, df = 2) (Fig. 2).

At the population level, we found that hexaploid populations produced fewer ovules (F = 15.51, *p* < 0.0001, df = 8) and had a higher P:O ratio compared to populations of other ploidy levels (F = 4.61, *p* < 0.001, df = 8) [see Additional file 6b,c]. However, tetraploid populations exhibited the most variation in pollen production, with the *Ei08* population having the highest values and the *Ei11* population having the lowest (F = 5.69, *p* < 0.0001, df = 8) [see Additional file 6a].

### Mating traits

**Fig. 3:**
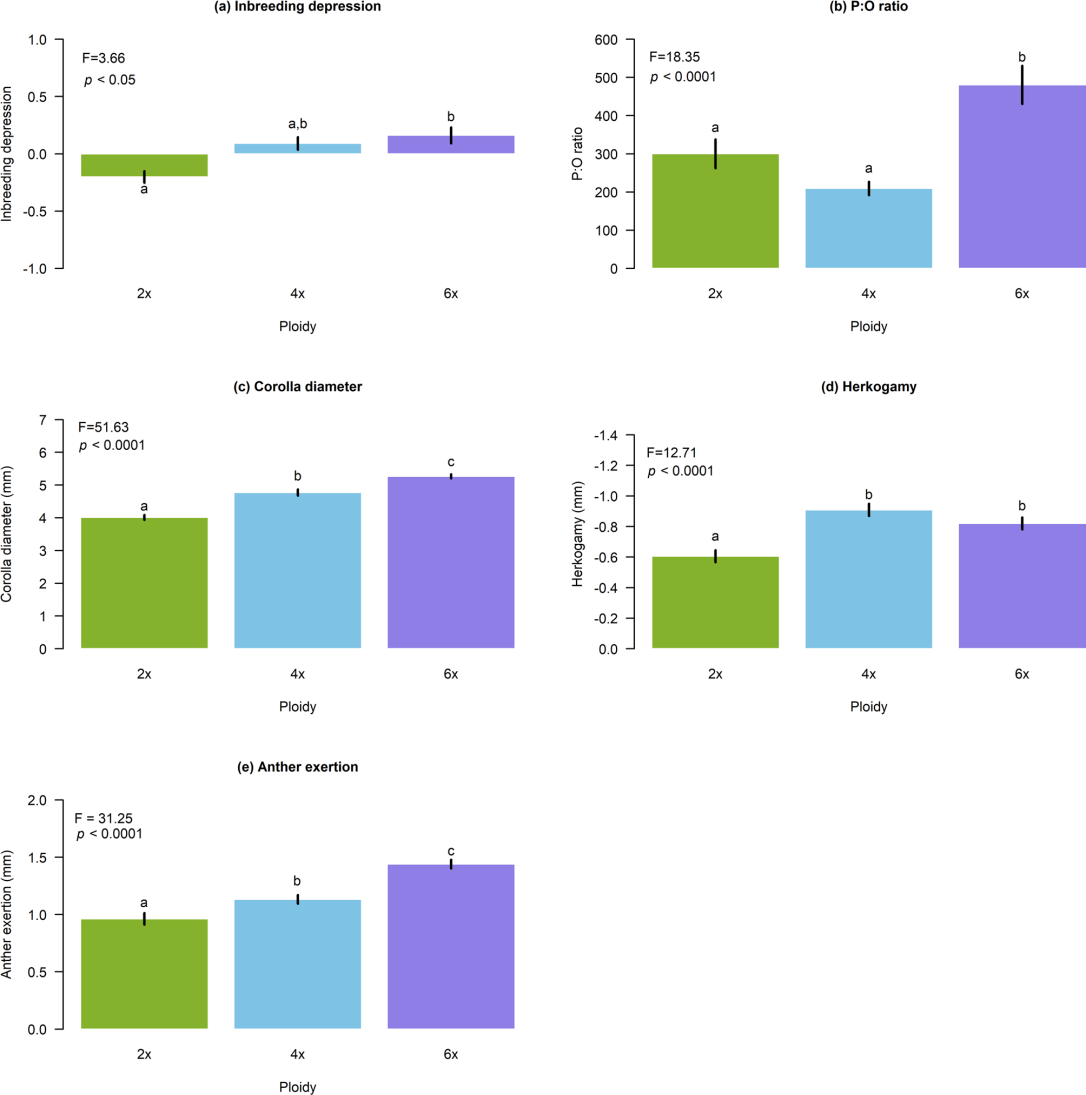
Mean values of traits related to the mating system: (**a)** inbreeding depression; (**b)** P:O ratio; and phenotypic traits: (**c)** corolla diameter, (**d)** herkogamy and (**e**) anther exertion measured in a group of 386 plants. Different letters indicate significant differences among ploidies according to the Tukey's test. Significance *p*-values indicate the ANOVA results among ploidies

Significant differences were observed in mating system traits for the group of 386 individuals from the three ploidy levels, encompassing all relevant traits. Inbreeding depression was positive in hexaploids and negative in diploids (Fig. 3a), as observed in the previous dataset of 759 individuals (see Inbreeding Depression section). Tetraploids showed intermediate inbreeding depression values, which were not significantly different from zero (F = 3.66, *p* < 0.05, df = 2) (Fig. 3a). The mean P:O ratio was significantly higher in hexaploids (F = 18.35, *p* < 0.0001, df = 2), while diploids and tetraploids showed lower and statistically similar P:O ratios (Fig. 3b). Floral traits showed significant differences among ploidy levels, with larger flowers observed as ploidy increased (F = 51.63, *p* < 0.0001, df = 2), and hexaploid plants exhibited the largest corolla sizes (Fig. 3c). Herkogamy was also greater in tetraploids and hexaploids (F = 12.71, *p* < 0.0001, df = 2), indicating more separation between sexual organs in polyploids (Fig. 3d). Anther exertion was significantly higher in polyploids (F = 31.25, *p* < 0.0001, df = 2), as polyploids exhibited greater anther exposure (Fig. 3e).

**Fig. 4:**
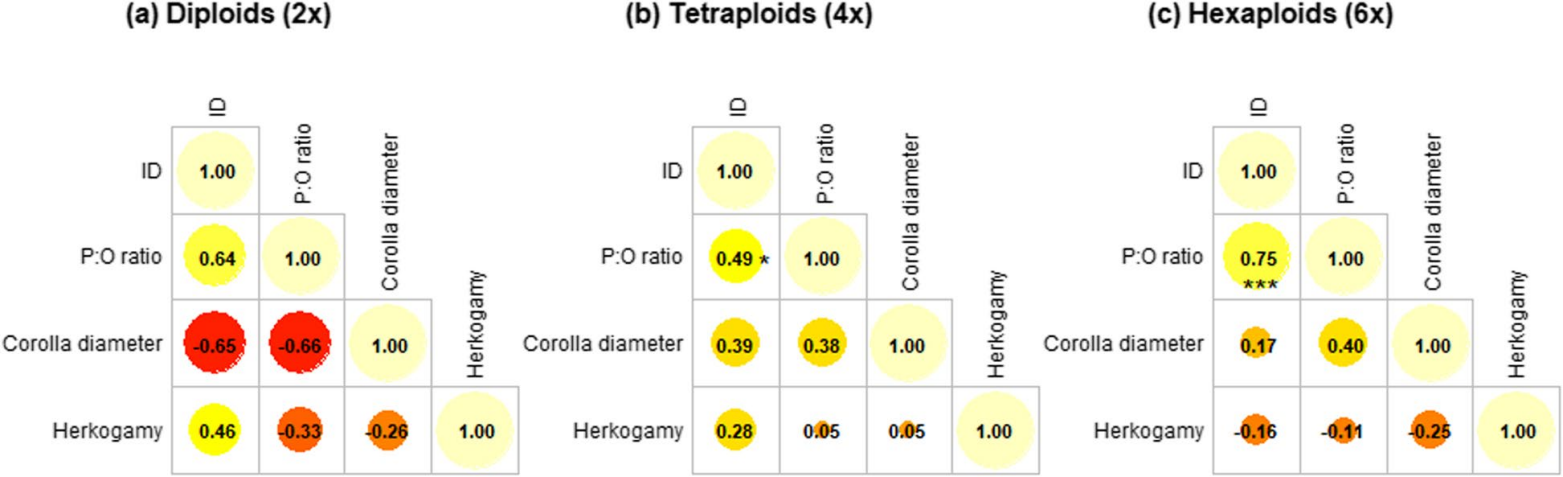
Correlation between pairs of mating traits across the three levels of ploidy: (**a**) diploids, (**b**) tetraploids and (**c**) hexaploids. Colors and circle size represent the magnitude and sign of correlation coefficients, with reddish colors indicating negative correlation coefficients and yellowish colors indicating positive correlation coefficients. The value of each correlation coefficient is indicated. Asterisks indicate significant correlations. *p-value < 0.05; **p-value < 0.01; ***p-value < 0.001; ****p-value < 0.0001

We found a significant positive correlation between the P:O ratio and inbreeding depression in tetraploids, and a stronger correlation in hexaploids (Fig. 4c). That is, plants with lower compatibility with their own pollen produced proportionally more pollen compared to ovules. Additionally, plants with a higher P:O ratio had larger flowers in hexaploids (Fig. 4). Although not significant, plants with a lower P:O ratio showed lower herkogamy, particularly in tetraploids (Fig. 4b). Inbreeding depression did not correlate significantly with corolla diameter for any ploidy (Fig. 4). However, we found a significant positive correlation between inbreeding depression and herkogamy in diploids (Fig. 4a), indicating that plants with higher inbreeding depression had less separation between sexual organs. Finally, herkogamy was negatively correlated with corolla diameter in hexaploids, suggesting that larger flowers had greater separation between sexual organs (Fig. 4c). Overall, the strongest relationships among mating traits were observed in hexaploids, where larger flowers, more accessible anthers, and higher levels of inbreeding depression were evident.

### Genomic diversity

Genetic diversity was significantly higher in polyploid plants compared to diploids (F = 65.5, *p* < 0.0001, df = 2). Polyploids exhibited higher heterozygosity levels than diploids, with tetraploid and hexaploid plants showing similar levels of genomic diversity. The proportion of observed heterozygotic sites was 0.350 and 0.383 for the two diploid populations. The mean proportion of observed heterozygotic sites found in the tetraploid populations was 0.585, ranging between 0.571 and 0.602, while it was 0.601 for hexaploid populations, ranging from 0.545 to 0.606 in hexaploid populations This value was not significantly different between the two levels of polyploidy.

## Discussion

In this study, we examined the variation in mating system-related traits across different ploidy levels of *Erysimum incanum*. The general trends observed — including smaller flowers, lower pollen production, minimal herkogamy, and a lack of inbreeding depression — align with the traditional characterization of *E. incanum* as a predominantly selfing plant [106]. However, we found a nuanced gradient of selfing tolerance that varied not only between ploidy levels but also among populations within the same ploidy. This variation ranged from clear signs of outbreeding depression to inbreeding depression, with several populations showing intermediate values (Table 3).

**Table 3:** Summary of the traits used for characterising the mating system of the three ploidies found in *E. incanum*. Note that here were refer to the absolute magnitude of herkogamy (this is, higher separation between anthers and stigma) since its value in *E. incanum* is always negative

Ploidy	Mating traits							Predicted mating system
	**Self-compatibility**	**Inbreeding depression**	**P:O ratio**	**Corolla size**	**Herkogamy**	**Anther exerion**	**Genetic diversity**	
2x	+ +	–	-	-	+	-	-	Selfing
4x	+	- +	+	+	+ +	+	+	Selfing-Outcrossing
6x	-	+	+ +	+ +	+ + +	+ +	+	Selfing-Outcrossing

Inbreeding depression is typically a key counterforce to selfing because of the negative effects of inbreeding [31]. Yet, paradoxically, it can also facilitate a shift toward inbreeding by purging deleterious alleles over successive generations of self-fertilization [84]. The presence of inbreeding depression in a selfing species like *E. incanum* is thus unexpected, especially since selfing species are thought to lack recessive deleterious alleles [12, 82, 131] but see [144]. Our findings underscore the success of outcrossing events, particularly in hexaploid populations, where we detected positive inbreeding depression. This result contradicts theoretical predictions that postulate a higher incidence of inbreeding depression in polyploids [18, 84]. In contrast, this suggests that polyploidy might increase genetic diversity — perhaps fortuitously, given the increased mutation rate [112] — and mask deleterious alleles, thus allowing some tolerance for selfing in the early generations [124]. On the other hand, selfing tolerance in diploid populations may be higher, as the purging of harmful alleles occurs more rapidly in diploids because they do not remain unmasked as they do in polyploids [127]. Our data suggest that in polyploid populations, outcrossing could mitigate the effects of deleterious allele accumulation, further supporting the notion that *E. incanum* exhibits outbreeding depression at lower ploidy levels.

Interestingly, inbreeding depression also varied at the population level. Previous research has shown similar population-specific variation in self-pollen tolerance, inbreeding depression, and outcrossing rates within a single species [61, 95, 121]. This variation highlights the importance of accounting for among-population differences when assessing the mating system of a species [140]. The observed patterns in *E. incanum* populations may reflect underlying ecological or genetic processes that shape mating strategies in different environments. As mating systems can fluctuate over time and space within a species, these findings invite further exploration of how mechanisms driving self-fertilization evolve in response to natural selection [74].

In this study, we assessed inbreeding depression through post-pollination fitness components, focusing on the interaction between pollen and stigma. We observed that both fertility and fertilization success decreased with increasing ploidy, with fertilization success being particularly diminished. This suggests that self-fertilization in *E. incanum* fails at the seed formation stage, even though initial studies did not reveal significant differences in the growth of self-pollen tubes on the stigma in vivo [see Additional File 7]. It is important to note that inbreeding depression can be evaluated across various life stages, and its magnitude may vary depending on the fitness component examined [74]. Previous studies have shown that inbreeding depression tends to be more pronounced in early stages of offspring development [71], but other research suggests it may be more variable across later life stages [58]. Future work examining other fitness components post-pollination could provide deeper insight into the long-term consequences for genetic diversity and population structure [17].

Ecological factors influencing outcrossing rates are well-documented [36, 100], but genetic factors, including ploidy, also play a role in shaping mating strategies [69, 111, 113]. Our results reveal that ploidy influences key traits related to pollination. Hexaploid *E. incanum* exhibited larger flowers, increased pollen production, and a higher P:O ratio, indicating a potential shift toward outcrossing. The higher pollen production in hexaploids could reflect an increased capacity for pollen exportation, rather than self-pollination alone. The P:O ratio has been shown to be a reliable predictor of reproductive strategies across various species [55, 61, 76, 93, 119] and continues to be a central concept in pollination research [65]. Interestingly, in the populations studied, plants with higher pollen production also tend to have larger flowers, which may attract more pollinators and facilitate pollen dispersal [26, 52]. This suggests that reproductive resources may be allocated jointly to primary (flowers) and secondary (pollen) sexual traits, enhancing the overall reproductive strategy.

Moreover, the observed levels of heterozygosity in polyploids may support the putative occurrence of outcrossing events. although these data are limited. Increasing genetic diversity is a significant consideration when studying species with contrasting mating systems. The fact that both tetraploid and hexaploid populations exhibited similar levels of heterozygosity suggests that this increase is not solely a result of genome duplication. Instead, it may reflect the impact of outcrossing events within these populations. Recent studies on polyploid species have emphasized the role of mating systems and ploidy in shaping genomic diversity and plant diversification [145]. In *E. incanum*, the observed rise in genetic diversity in higher ploidy levels is noteworthy, particularly since inbred populations typically experience reduced genetic diversity [72, 132]. Our findings indicate that polyploidy may facilitate a shift toward outcrossing in some populations, providing further evidence that ploidy and mating system dynamics can jointly influence species diversification, as seen in previous studies [145]. However, a higher number of samples is needed to find stronger evidence. While the origin of polyploid populations (autopolyploid or allopolyploid) remains unclear, determining this will be crucial to understanding the inter-population variation observed in polyploids.

Herkogamy, the spatial separation of sexual organs, is a key trait that influences selfing and outcrossing rates, [75, 136]. Along with flower size, herkogamy has been used as a reliable indicator of a species'mating system, as demonstrated in *Clarkia* species [19, 48]. In *E. incanum*, we observed that species with smaller flowers and reduced herkogamy were more likely to self-pollinate, whereas those with larger flowers and greater herkogamy showed higher rates of outcrossing. These patterns suggest that flower size and herkogamy are genetically correlated, with indirect selection playing a significant role in their evolution. Larger flowers and greater herkogamy can reduce the likelihood of self-pollination, especially in populations with strong inbreeding depression, such as hexaploid populations. In these populations, following an outcrossing strategy would be beneficial for reproduction, due to pollinator attraction and pollen exportation may be favoured by the traits shown by these plants.

Our study also revealed significant correlations between mating traits, such as flower size, reproductive investment, and self-pollen tolerance. Previous studies have documented genetic correlations between morphological and reproductive traits [13, 128, 143], though this is not always the case [98]. In *E. incanum*, the variation in self-pollen tolerance across hexaploid populations likely accounts for some of the inconsistencies in these correlations. Nonetheless, the correlation between the P:O ratio and inbreeding depression remained consistent across ploidy levels, supporting the idea that the P:O ratio is a reliable indicator of reproductive strategy [42]. Interestingly, in tetraploids, the correlation between inbreeding depression and corolla size suggests that larger flowers are more likely to undergo cross-pollination. Similar patterns have been observed in other *Erysimum* species [3], where increased flower size correlates with higher levels of inbreeding depression in a generalist pollinated plant. In fact, in other species, it has been shown that morphological changes associated with increased ploidy can significantly alter the ecological interactions experienced by plants [116],and the references therein) and, by extension, modify rates of outcrossing.

## Conclusions

In summary, our study revealed considerable variation in self-pollen tolerance among different ploidy levels and populations of *E. incanum*, challenging the notion that the species is predominantly self-compatible. As ploidy increases, self-compatibility decreases, accompanied by shifts in floral traits such as flower size, reproductive investment, and herkogamy. Diploid populations appear to exhibit a clear selfing strategy with significant outcrossing depression, while hexaploid plants show substantial inbreeding depression and larger flowers, higher herkogamy, and a higher P:O ratio. These results emphasize the importance of accounting for intraspecific variation when characterizing the mating systems of plant species, particularly in understanding how ploidy may drive shifts toward alternative reproductive strategies, which can significantly influence population genetics, genome structure, and ecological interactions. Ultimately, these shifts may contribute to the evolutionary transition toward outcrossing, a process that could play a crucial role in speciation.

## Supplementary Information

Additional file 1. Somatic chromosomes of Erysimum incanum visualized in root meristem cells. A) View of chromosomes of E. incanum ssp. mairei (2n = 16); B) of E. incanum ssp. incanum (2n = 32); C) and of E. incanum from the High Atlas and Anti-Atlas Mountains (2n = 48). Adapted from Abdelaziz et al. (In prep.).

Additional file 2. Mean values of fitness components estimated from (a) selfing and (b) outcrossing treatments for each ploidy. The fitness components were seedset, measured as the proportion of filled seeds by the total ovule production; fertility, as the proportion of fertilised ovules (seeds and aborts) by the total ovule production; and fertility success, as the proportion of filled seeds by the total number of fertilised ovules. Different letters indicate significant differences among ploidies according to the Tukey's test. Significance *p*-values indicate the ANOVA results among ploidies (n.s = non-significant).

Additional file 3. Outcome of the GLMM testing the effect of the treatment and the ploidy as fixed factors together with their interaction on E. incanum fertility. The individual plant and the population appear as random factors nested within the ploidy level. Significance p-values are indicated in bold (*p-value < 0.05; **p-value < 0.01; ***p-value < 0.001; ****p-value < 0.0001).

Additional file 4. Outcome of the GLMM testing the effect of the treatment and the ploidy as fixed factors together with their interaction on E. incanum fertilization success. The individual plant and the population appear as random factors nested within the ploidy level. Significance p-values are indicated in bold (*p-value < 0.05; **p-value < 0.01; ***p-value < 0.001; ****p-value < 0.0001).

Additional file 5. Mean values of seedset estimated from (a) selfing and (b) outcrossing treatments for each population. Green bars refer to diploid, blue bars to tetraploid, and purple bars to hexaploid populations. Different letters indicate significant differences among ploidies according to the Tukey's test. Significance p-values indicate the ANOVA results among ploidies (n.s = non-significant)

Additional file 6. Reproductive investment among populations for (a) male function measured as pollen production, (b) female function measured as ovule amount and (c) the relative investment between male and female function estimated as P:O ratio. Green bars refer to diploid, blue bars to tetraploid, and purple bars to hexaploid populations. Significance p-values indicate the ANOVA results among ploidies

Additional file 7. Visualisation of pollen tube growth in vivo experiments. Pollen tube growth on the stigma of a self-pollinated flower from diploid, tetraploid and hexaploid plants was visualised by UV microscopy. Self-pollinated flowers were preserved in alcohol 90º for 72 hours and were subjected to an aniline blue staining protocol modified from Xie et al. (2017).

Additional file 8. Mathematical notation corresponding to each model. Models were tested with lme4 package in R for the effect of the independent variables Treatment and Ploidy and their interaction on the dependent variables Seedset, Fertility and Fertilization success. The terms β are the coefficient associated to each independent variable, 𝑏 is the random effect and 𝑒 is the random error for the 𝑖 plant and the 𝑗 observation.

## Data Availability

The datasets generated and used in this study are stored for review purposes at https://drive.google.com/drive/u/0/folders/1I0b6PWgGriyFo3XtEh45qE5Gx6Yjcsc6, and will be made available in a public repository such as Zenodo upon manuscript acceptance.
