# Supplementary material for: Can ploidy changes propel the evolution of allogamy in a selfing species complex?
Source: BMC Plant Biol. 2025 Aug 1;25:1011. doi: 10.1186/s12870-025-06868-1 (PMC12315261; doi:10.1186/s12870-025-06868-1)

Additional File 1: Figure S1. Somatic chromosomes of *Erysimum incanum* visualized in root meristem cells. A) View of chromosomes of *E. incanum* ssp. *mairei* (2n = 16); B) of *E. incanum* ssp. *incanum* (2n = 32); C) and of *E. incanum* from the High Atlas and Anti-Atlas Mountains (2n = 48). Adapted from Abdelaziz et al. (*In prep.*).


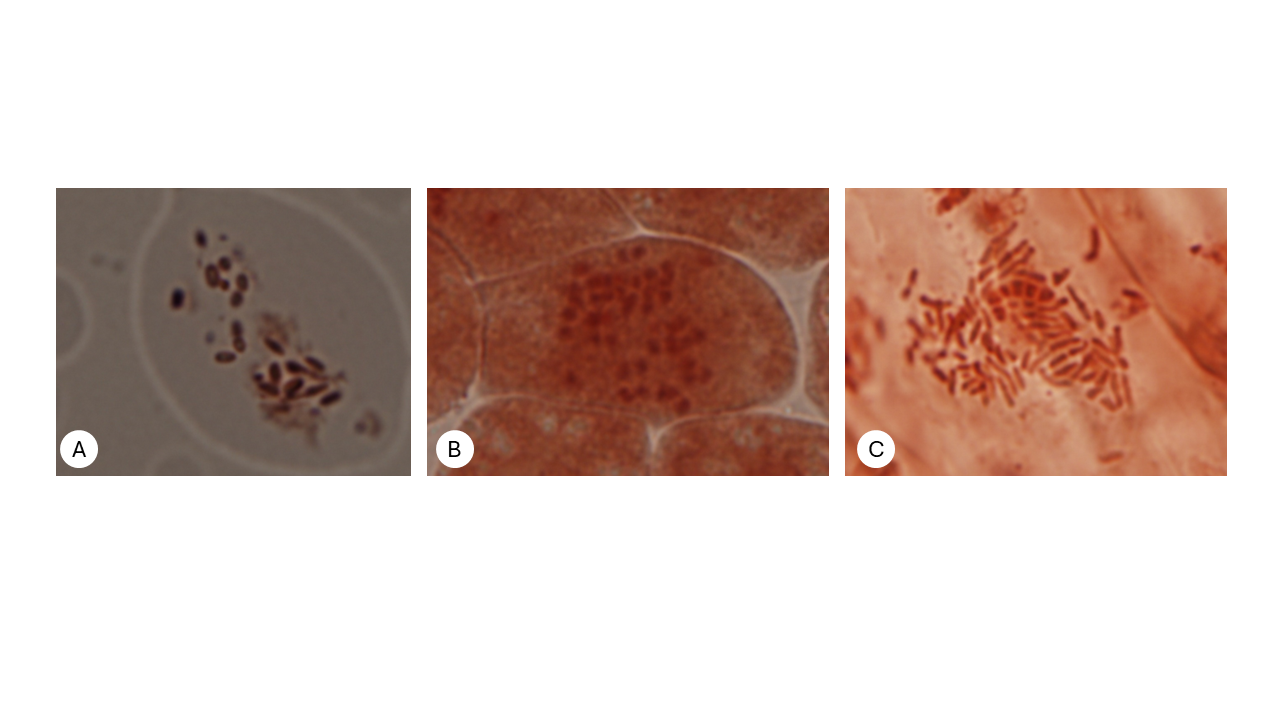

Supplement: Supplementary file 1 — Additional file 1. Somatic chromosomes of Erysimum incanum visualized in root meristem cells. A) View of chromosomes of E. incanum ssp. mairei (2n = 16); B) of E. incanum ssp. incanum (2n = 32); C) and of E. incanum from the High Atlas and Anti-Atlas Mountains (2n = 48). Adapted from Abdelaziz et al. (In prep.). [file 12870_2025_6868_MOESM1_ESM.docx]
