# Supplementary material for: Can ploidy changes propel the evolution of allogamy in a selfing species complex?
Source: BMC Plant Biol. 2025 Aug 1;25:1011. doi: 10.1186/s12870-025-06868-1 (PMC12315261; doi:10.1186/s12870-025-06868-1)

Additional file 2. Mean values of fitness components estimated from (a) selfing and (b) outcrossing treatments for each ploidy. The fitness components were seedset, measured as the proportion of filled seeds by the total ovule production; fertility, as the proportion of fertilised ovules (seeds and aborts) by the total ovule production; and fertilizaation success, as the proportion of filled seeds by the total number of fertilised ovules. Different letters indicate significant differences among ploidies according to the Tukey's test. Significance *p*-values indicate the ANOVA results among ploidies (n.s = non-significant).


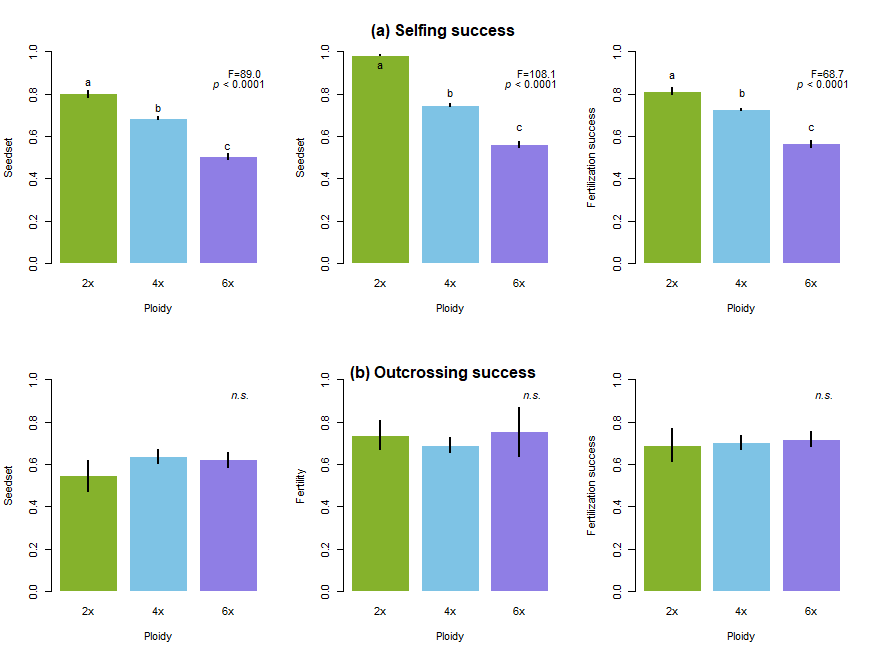

Supplement: Supplementary file 2 — Additional file 2. Mean values of fitness components estimated from (a) selfing and (b) outcrossing treatments for each ploidy. The fitness components were seedset, measured as the proportion of filled seeds by the total ovule production; fertility, as the proportion of fertilised ovules (seeds and aborts) by the total ovule production; and fertility success, as the proportion of filled seeds by the total number of fertilised ovules. Different letters indicate significant differences among ploidies according to the Tukey's test. Significance p-values indicate the ANOVA results among ploidies (n.s = non-significant). [file 12870_2025_6868_MOESM2_ESM.docx]
