# Supplementary material for: Can ploidy changes propel the evolution of allogamy in a selfing species complex?
Source: BMC Plant Biol. 2025 Aug 1;25:1011. doi: 10.1186/s12870-025-06868-1 (PMC12315261; doi:10.1186/s12870-025-06868-1)
Supplement: Supplementary file 4 — Additional file 4. Outcome of the GLMM testing the effect of the treatment and the ploidy as fixed factors together with their interaction on E. incanum fertilization success. The individual plant and the population appear as random factors nested within the ploidy level. Significance p-values are indicated in bold (*p-value < 0.05; **p-value < 0.01; ***p-value < 0.001; ****p-value < 0.0001). [file 12870_2025_6868_MOESM4_ESM.docx]

Additional file 4. Outcome of the GLMM testing the effect of the treatment and the ploidy as fixed factors together with their interaction on *E. incanum* fertilization success. The individual plant and the population appear as random factors nested within the ploidy level. Significance p-values are indicated in bold (*p-value < 0.05; **p-value < 0.01; ***p-value < 0.001; ****p-value < 0.0001).

|  |  | Fertilitization success | | | | | |
| --- | --- | --- | --- | --- | --- | --- | --- |
| Model | *Estimate* | AIC | BIC | logLik | *χ^2^* | df | *p-*value |
| Intercept | 0.60 | 3307.5 | 3326.8 | -1650.8 |  |  |  |
| Fertilization success~ Treatment + (1 \|Population/Plant) |  | 3297.9 | 3323.8 | -1645.0 | 11.555 | 1 | **<0.0001****** |
| *Outcrossing* | 0.09 |  |  |  |  |  |  |
| Fertilization success ~ Ploidy + (Ploidy \|Population/Plant) |  | 3276.8 | 3341.4 | -1628.4 | 33.116 | 11 | **<0.0001****** |
| *4x* | -0.23 |  |  |  |  |  |  |
| *6x* | -0.22 |  |  |  |  |  |  |
| Fertilization success ~ Treatment * Ploidy + (Ploidy \|Population/Plant) |  | 3257.6 | 3341.5 | -1615.8 | 25.261 | 3 | **<0.0001****** |
| *Outcrossing* | -0.12 |  |  |  |  |  |  |
| *4x* | -0.26 |  |  |  |  |  |  |
| *6x* | -0.30 |  |  |  |  |  |  |
| *Outcrossing:4x* | 0.18 |  |  |  |  |  |  |
| *Outcrossing:6x* | 0.32 |  |  |  |  |  |  |
