# Supplementary material for: Can ploidy changes propel the evolution of allogamy in a selfing species complex?
Source: BMC Plant Biol. 2025 Aug 1;25:1011. doi: 10.1186/s12870-025-06868-1 (PMC12315261; doi:10.1186/s12870-025-06868-1)

Additional file 5. Mean values of seedset estimated from (a) selfing and (b) outcrossing treatments for each population. Green bars refer to diploid, blue bars to tetraploid, and purple bars to hexaploid populations. Different letters indicate significant differences among ploidies according to the Tukey's test. Significance *p*-values indicate the ANOVA results among ploidies (n.s = non-significant).


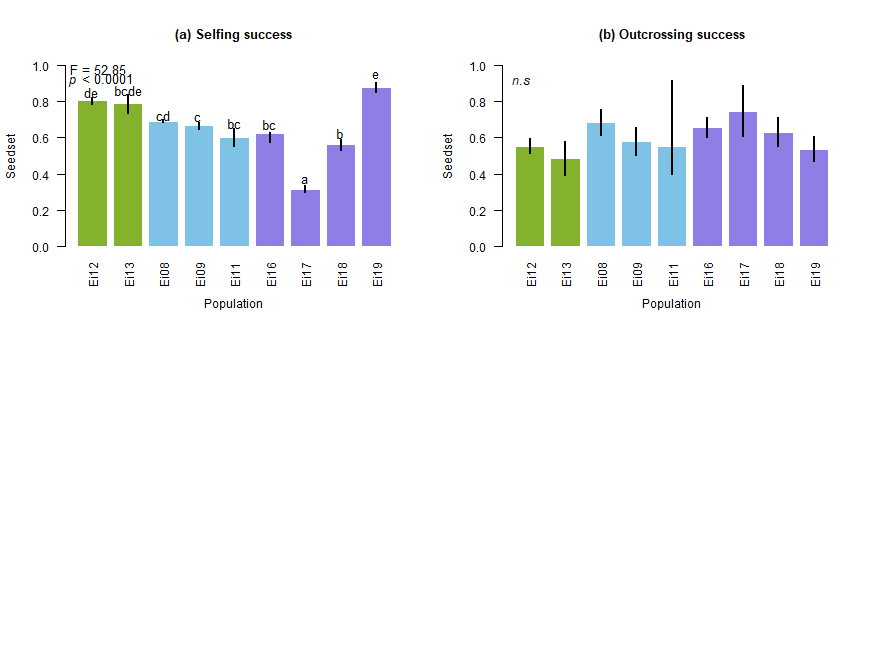

Supplement: Supplementary file 5 — Additional file 5. Mean values of seedset estimated from (a) selfing and (b) outcrossing treatments for each population. Green bars refer to diploid, blue bars to tetraploid, and purple bars to hexaploid populations. Different letters indicate significant differences among ploidies according to the Tukey's test. Significance p-values indicate the ANOVA results among ploidies (n.s = non-significant) [file 12870_2025_6868_MOESM5_ESM.docx]
