# Supplementary material for: Can ploidy changes propel the evolution of allogamy in a selfing species complex?
Source: BMC Plant Biol. 2025 Aug 1;25:1011. doi: 10.1186/s12870-025-06868-1 (PMC12315261; doi:10.1186/s12870-025-06868-1)

Additional file 6. Reproductive investment among populations for (a) male function measured as pollen production, (b) female function measured as ovule amount and (c) the relative investment between male and female function estimated as P:O ratio. Green bars refer to diploid, blue bars to tetraploids and purple bars to hexaploid populations. Significance *p*-values indicate the ANOVA results among ploidies.


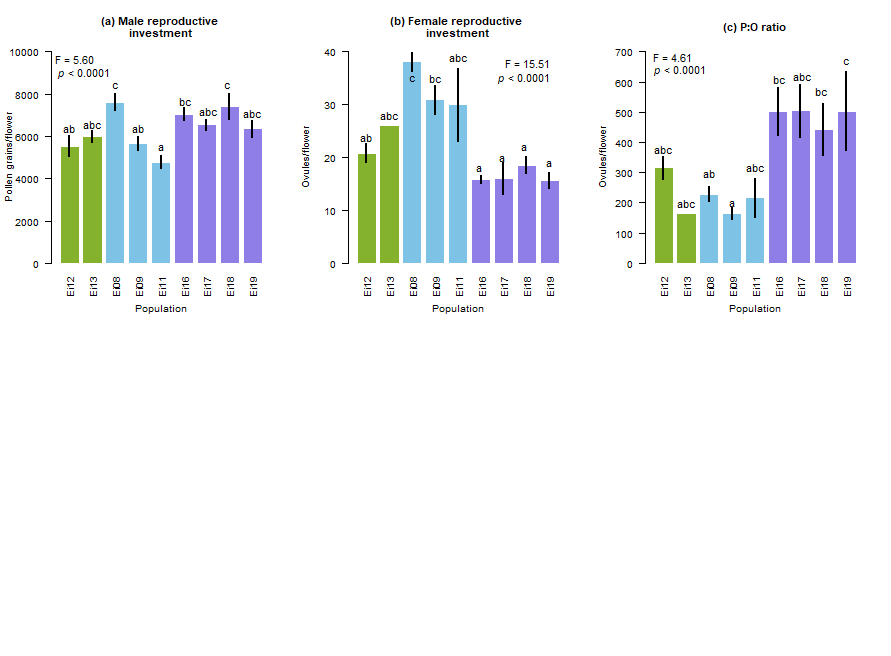

Supplement: Supplementary file 6 — Additional file 6. Reproductive investment among populations for (a) male function measured as pollen production, (b) female function measured as ovule amount and (c) the relative investment between male and female function estimated as P:O ratio. Green bars refer to diploid, blue bars to tetraploid, and purple bars to hexaploid populations. Significance p-values indicate the ANOVA results among ploidies [file 12870_2025_6868_MOESM6_ESM.docx]
