# Supplementary material for: Can ploidy changes propel the evolution of allogamy in a selfing species complex?
Source: BMC Plant Biol. 2025 Aug 1;25:1011. doi: 10.1186/s12870-025-06868-1 (PMC12315261; doi:10.1186/s12870-025-06868-1)

Additional File 7. Visualisation of pollen tube growth in vivo experiments. Pollen tube growth on the stigma of a self-pollinated flower from diploid, tetraploid and hexaploid plants was visualised by UV microscopy. Self-pollinated flowers were preserved in alcohol 90º for 72 hours and were subjected to an aniline blue staining protocol modified from [Xie et al. (2017)](https://paperpile.com/c/XvY2ud/DivA).


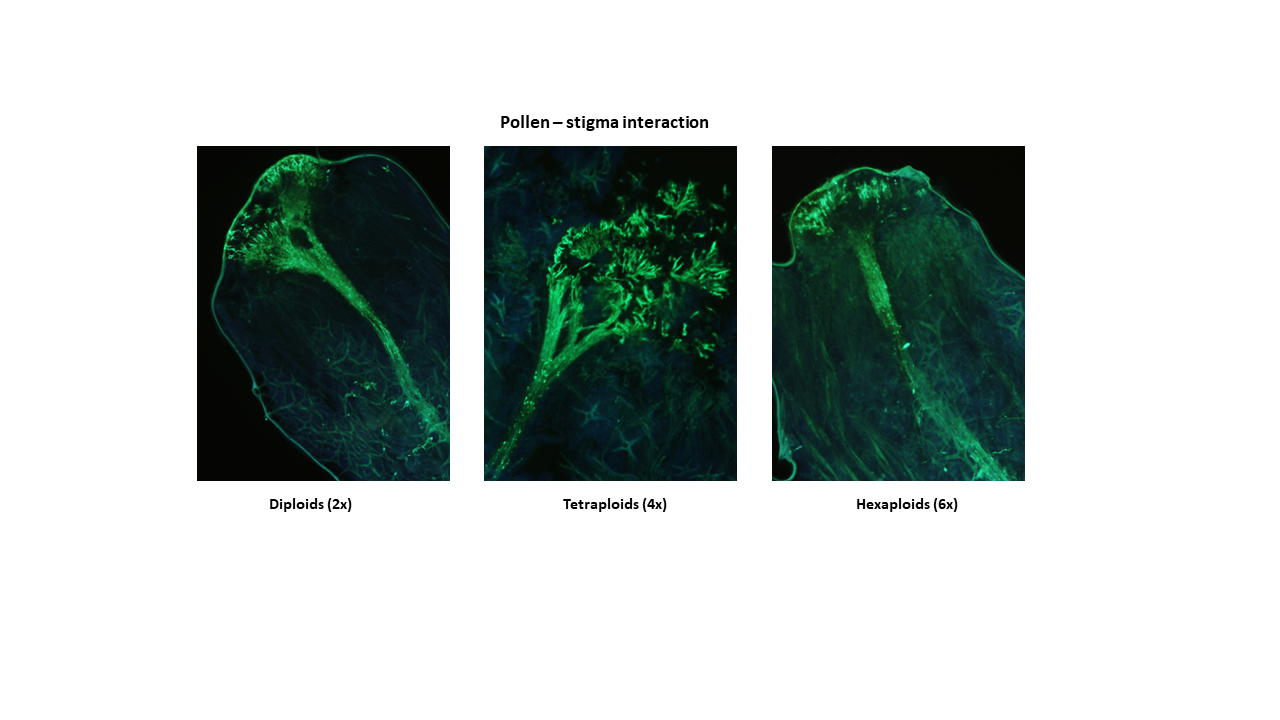

Supplement: Supplementary file 7 — Additional file 7. Visualisation of pollen tube growth in vivo experiments. Pollen tube growth on the stigma of a self-pollinated flower from diploid, tetraploid and hexaploid plants was visualised by UV microscopy. Self-pollinated flowers were preserved in alcohol 90º for 72 hours and were subjected to an aniline blue staining protocol modified from Xie et al. (2017). [file 12870_2025_6868_MOESM7_ESM.docx]
