# Supplementary material for: Can ploidy changes propel the evolution of allogamy in a selfing species complex?
Source: BMC Plant Biol. 2025 Aug 1;25:1011. doi: 10.1186/s12870-025-06868-1 (PMC12315261; doi:10.1186/s12870-025-06868-1)
Supplement: Supplementary file 8 — Additional file 8. Mathematical notation corresponding to each model. Models were tested with lme4 package in R for the effect of the independent variables Treatment and Ploidy and their interaction on the dependent variables Seedset, Fertility and Fertilization success. The terms β are the coefficient associated to each independent variable, 𝑏 is the random effect and 𝑒 is the random error for the 𝑖 plant and the 𝑗 observation. [file 12870_2025_6868_MOESM8_ESM.docx]

Additional File 8. Mathematical notation corresponding to each model. Models were tested with *lme4* package in R for the effect of the independent variables *Treatment* and *Ploidy* and their interaction on the dependent variables *Seedset, Fertility* and *Fertilization success.* The terms β are the coefficient associated to each independent variable, 𝑏 is the random effect and 𝑒 is the random error for the 𝑖 plant and the 𝑗 observation.

| *lm4* package notation |  | | Mathematical notation | |
| --- | --- | --- | --- | --- |
| Intercept |  | ${Seedset}_{ij}=\beta_{0}+b_{0i}+ e_{ij}$ | |  |
| Seedset ~ Treatment + (1 \|Population/Plant) |  | ${Seedset}_{ij}=\beta_{0}{+ \beta}_{1}\cdot{Treatment}_{ij}+b_{0i}+ e_{ij}$ | |  |
| Seedset ~ Ploidy + (Ploidy \|Population/Plant) |  | ${Seedset}_{ij}=\beta_{0}{+ \beta}_{1}\cdot{Ploidy}_{ij}+b_{0i}+b_{li}\cdot{Ploidy}_{ij}+ e_{ij}$ | |  |
| Seedset ~ Treatment * Ploidy + (Ploidy \|Population/Plant) |  | ${Seedset}_{ij}=\beta_{0}{+ \beta}_{1}\cdot{Treatment}_{ij}{+ \beta}_{2}\cdot{Ploidy}_{ij}{+ \beta}_{3}\cdot{({Treatment}_{ij}\times Ploidy}_{ij})+b_{0i}+b_{li}\cdot{Ploidy}_{ij}+ e_{ij}$ | |  |
|  |  |  | |  |
| Intercept |  | ${Fertility}_{ij}=\beta_{0}+b_{0i}+ e_{ij}$ | |  |
| Fertility ~ Treatment + (1 \|Population/Plant) |  | ${Fertility}_{ij}=\beta_{0}{+ \beta}_{1}\cdot{Treatment}_{ij}+b_{0i}+ e_{ij}$ | |  |
| Fertility ~ Ploidy + (Ploidy \|Population/Plant) |  | ${Fertility}_{ij}=\beta_{0}{+ \beta}_{1}\cdot{Ploidy}_{ij}+b_{0i}+b_{li}\cdot{Ploidy}_{ij}+ e_{ij}$ | |  |
| Fertility ~ Treatment * Ploidy + (Ploidy \|Population/Plant) |  | ${Fertility}_{ij}=\beta_{0}{+ \beta}_{1}\cdot{Treatment}_{ij}{+ \beta}_{2}\cdot{Ploidy}_{ij}{+ \beta}_{3}\cdot{({Treatment}_{ij}\times Ploidy}_{ij})+b_{0i}+b_{li}\cdot{Ploidy}_{ij}+ e_{ij}$ | |  |
|  |  |  | |  |
| Intercept |  | ${Seedset}_{ij}=\beta_{0}+b_{0i}+ e_{ij}$ | |  |
| Fertilization success ~ Treatment + (1 \|Population/Plant) |  | ${Seedset}_{ij}=\beta_{0}{+ \beta}_{1}\cdot{Treatment}_{ij}+b_{0i}+ e_{ij}$ | |  |
| Fertilization success ~ Ploidy + (Ploidy \|Population/Plant) |  | ${Seedset}_{ij}=\beta_{0}{+ \beta}_{1}\cdot{Ploidy}_{ij}+b_{0i}+b_{li}\cdot{Ploidy}_{ij}+ e_{ij}$ | |  |
| Fertilization success ~ Treatment * Ploidy + (Ploidy \|Population/Plant) |  | ${Seedset}_{ij}=\beta_{0}{+ \beta}_{1}\cdot{Treatment}_{ij}{+ \beta}_{2}\cdot{Ploidy}_{ij}{+ \beta}_{3}\cdot{({Treatment}_{ij}\times Ploidy}_{ij})+b_{0i}+b_{li}\cdot{Ploidy}_{ij}+ e_{ij}$ | |  |
